# Point-of-Care Ultrasound for Traumatic Diaphragmatic Hernia in a Low- and Middle-Income Country: A Case Report

**DOI:** 10.7759/cureus.87630

**Published:** 2025-07-09

**Authors:** Kwabena A Danso, Kwame Ekremet, Sheba Fiadzomor

**Affiliations:** 1 Emergency Medicine, Komfo Anokye Teaching Hospital, Kumasi, GHA; 2 Emergency Medicine, University of Ghana Medical Centre, Accra, GHA; 3 Emergency Medicine, 37 Military Hospital, Accra, GHA

**Keywords:** blunt injury, diaphragmatic hernia, lmic, pocus, traumatic diaphragmatic hernia

## Abstract

This case report highlights the successful use of point-of-care ultrasound (POCUS) in a low- and middle-income country (LMIC) to achieve early diagnosis of a traumatic diaphragmatic hernia (DH), leading to timely surgical intervention. Traumatic DH, often resulting from blunt trauma, poses a diagnostic challenge due to its rarity, potential for occult presentation, and the presence of other life-threatening injuries that may obscure its detection. Delayed diagnosis can result in significant mortality, primarily due to cardiorespiratory compromise from visceral herniation. This report underscores the value of bedside ultrasound in resource-limited EDs as a critical tool for evaluating traumatic DH during the initial assessment, with the potential to improve patient outcomes in such settings. To the best of our knowledge, this is the first reported case in an LMIC in which POCUS played a pivotal role in the timely diagnosis and management of this condition.

## Introduction

The trunk of the human body is divided into the abdominal and thoracic cavities by a C-shaped, thin, musculo-tendinous structure known as the diaphragm. The diaphragm is the primary muscle of respiration, maintaining negative pressure within the thoracic cavity and positive pressure within the abdomen [1]. Traumatic diaphragmatic hernia (DH) may result from either blunt or penetrating injuries, with delayed diagnosis often attributed to a focus on other life-threatening injuries, absence of specific clinical signs, limited access to advanced imaging modalities, or simply a failure to consider the diagnosis [2,3]. A study by Fair et al. identified traumatic DH as an uncommon condition that is often caused by blunt injury mechanisms and may present occultly [4]. Notably, over 80-90% of diaphragmatic injuries occur on the left leaflet of the diaphragm [5,6]. According to studies, the mortality rate following diaphragmatic rupture ranges from 17% to 21%, with the primary cause of death being cardiorespiratory compromise resulting from visceral herniation into the thoracic cavity through the diaphragm [1,4].

To the best of our knowledge, this is the first reported case from a low- and middle-income country (LMIC) where POCUS facilitated the early diagnosis of a traumatic DH, leading to prompt surgical intervention. The aim of this case report is to highlight the utility of bedside ultrasound in EDs within resource-limited healthcare settings for the evaluation of traumatic DH during initial patient assessment.

## Case presentation

A 13-year-old boy suffered blunt chest and abdominal trauma on February 2, 2025, after the weight of sand and earth from a dug hole collapsed on him while he was playing with friends in a pit. He was rushed to a nearby healthcare facility for initial management, which included administration of analgesics and oxygen support; however, no imaging was performed. He was subsequently referred to the ED for imaging and definitive care. The patient complained of chest pain associated with difficulty in breathing.

At presentation, he was conscious but confused and in evident respiratory distress, as demonstrated by nasal flaring, difficulty completing sentences, and a rapid respiratory rate (>26 cycles per minute). He was tachycardic (105 bpm), with a Glasgow Coma Score of 14 (M6V4E4). Chest examination revealed asymmetry, with the left side slightly elevated compared to the right. No bruising or contusions were noted on the skin. Breath sounds and air entry were reduced on the left hemithorax. His oxygen saturation was 94% on a non-rebreather mask delivering oxygen at 15 L/min. His lips and oral mucosa appeared progressively drier with each labored breath.

**Figure 1:**
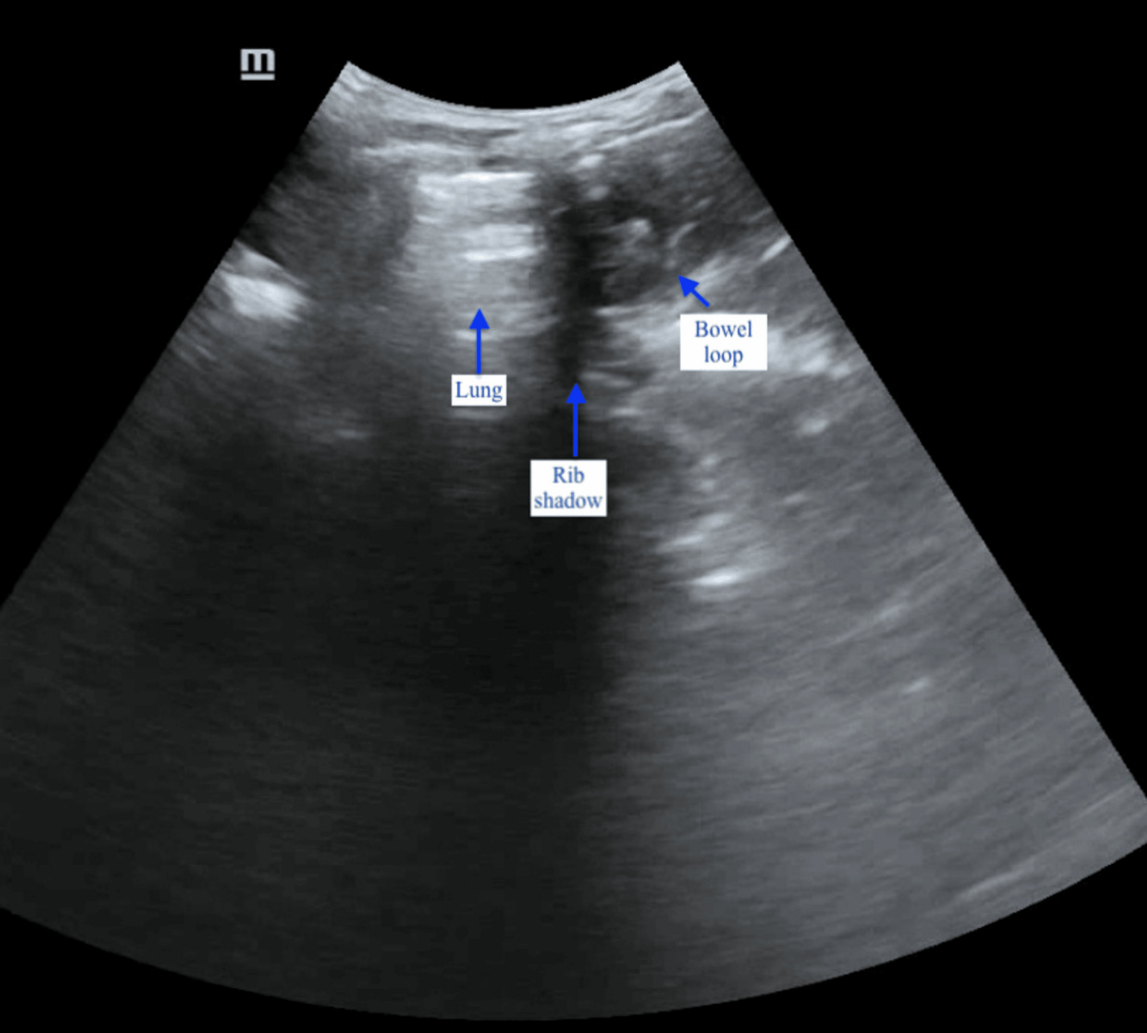
Ultrasound image of the left lung parenchyma (showing A-lines) on the left side of the rib shadow, with a bowel loop visible on the right side The probe marker was oriented toward the patient’s head.

**Figure 2:**
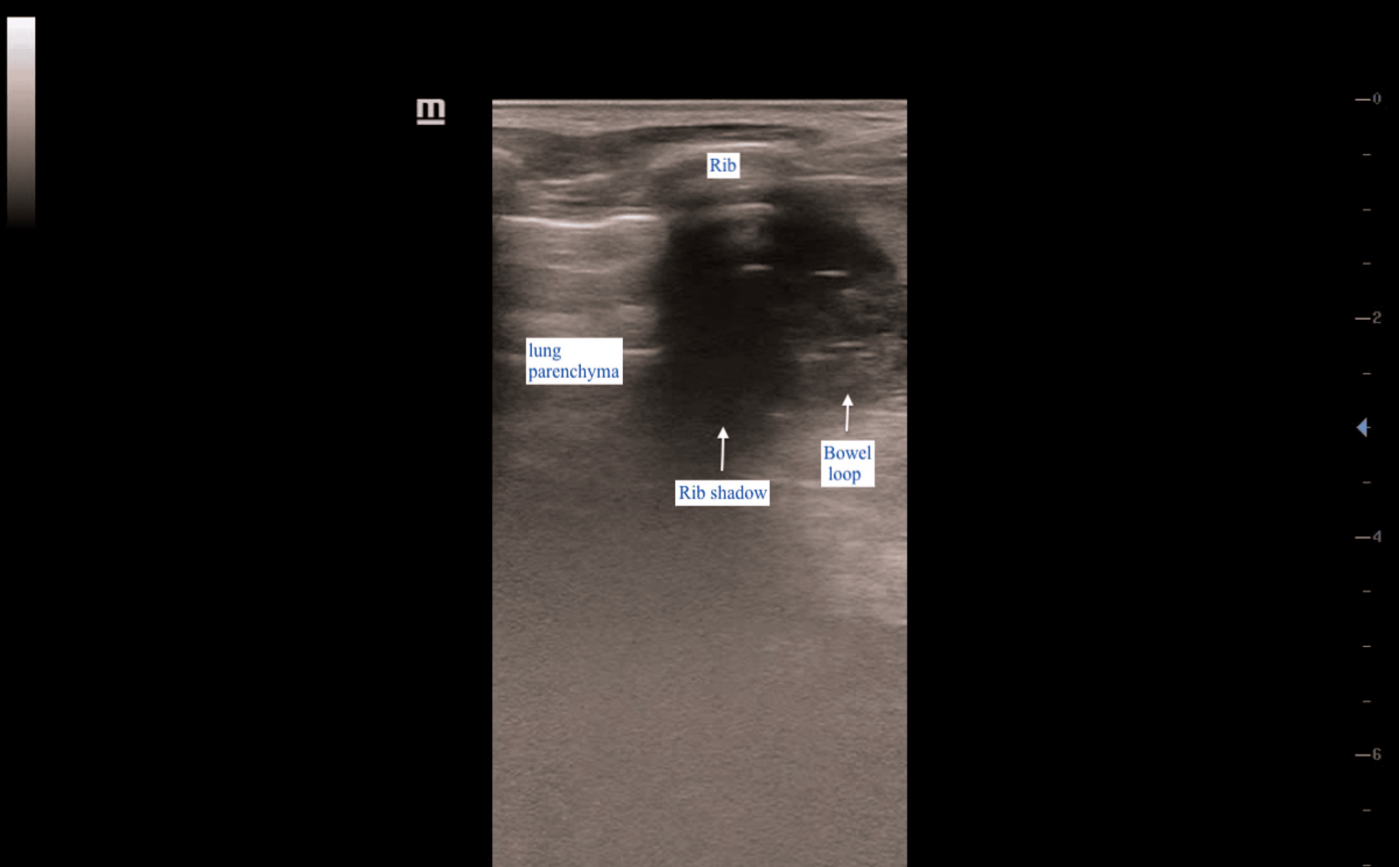
Linear probe image showing the lung and bowel loop in close proximity The diaphragm, spleen, and left kidney are absent. The left side of the image corresponds to the patient’s head.

A rapid bedside ultrasound scan was performed to evaluate for the presence of free fluid and air in the pleural, abdominal, and pericardial spaces as part of the extended focused assessment with sonography in trauma. Point-of-care ultrasound (POCUS) findings were unremarkable in the right upper quadrant but inconclusive on the left, where visualization of the left kidney and spleen was poor. Further scanning of the left upper quadrant - between the mid- and posterior axillary lines and extending cephalad - showed that the left hemidiaphragm could not be visualized using both the curvilinear (3.5 MHz) and linear (7.5 MHz) probes. Additionally, bowel loops with peristaltic activity were observed in close proximity to the left lung field (Figure 1, Figure 2).

**Figure 3:**
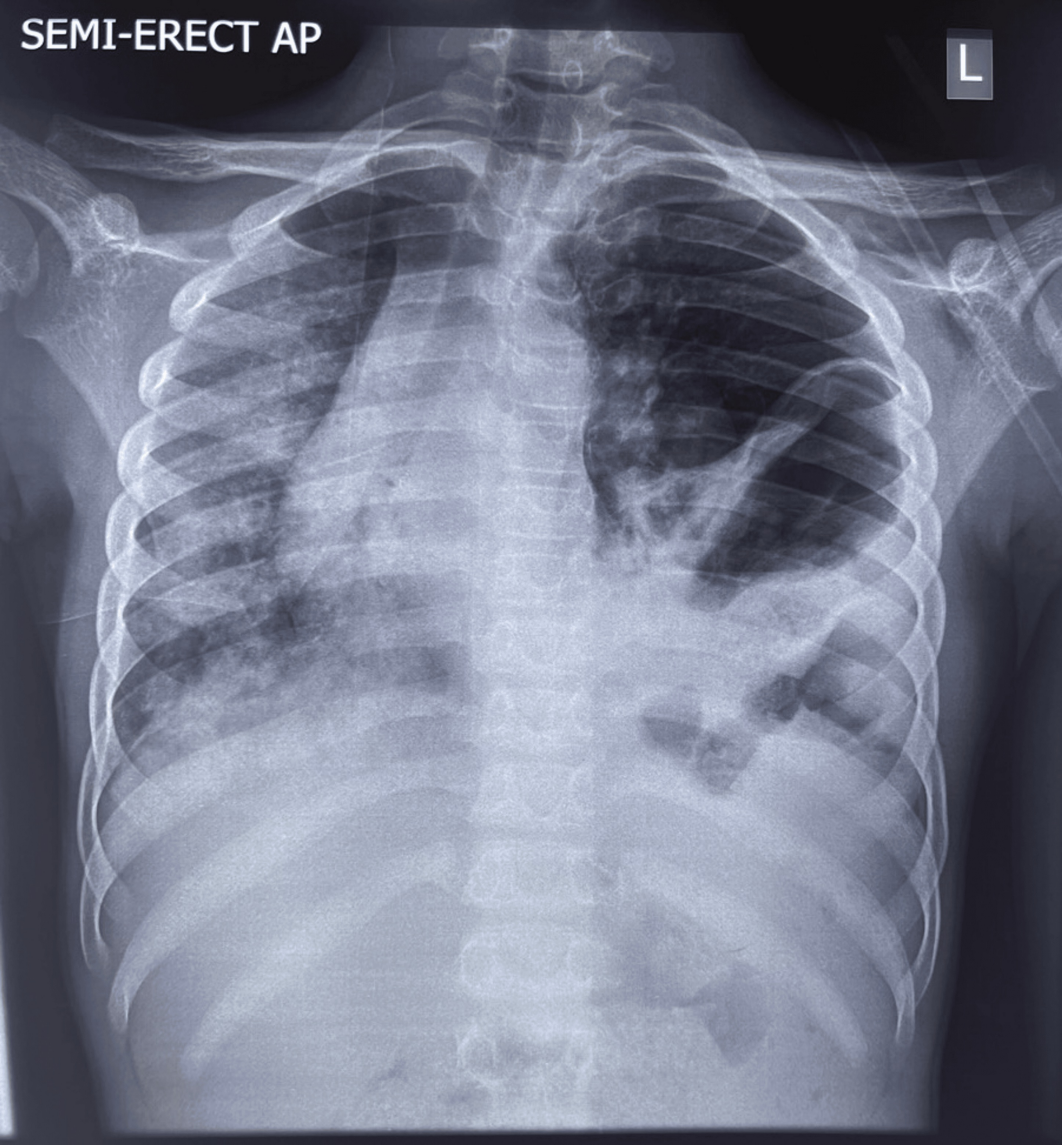
Semi-erect chest X-ray showing rightward shift of the trachea and mediastinum, absent lung markings on the left side, and a disrupted left hemidiaphragm

There was no lung sliding observed on the anterior chest wall of the left hemithorax when assessed with the linear probe. The patient was resuscitated, and a chest radiograph was subsequently performed (Figure 3).

Chest radiography revealed deviation of the trachea and right main bronchus to the right, absent lung markings on the left hemithorax, and disruption along the expected contour of the left hemidiaphragm, with an absent or poorly defined gastric bubble. Nasogastric tube insertion was attempted but was unsuccessful. The surgical team was contacted, informed consent was obtained and signed by the patient’s father, and the patient was scheduled for exploratory surgery and repair.

Intraoperative findings revealed a 12.5 cm tear in the left hemidiaphragm, with the stomach, transverse colon, and spleen herniated through the defect. These findings confirmed the initial suspicion of traumatic left diaphragmatic rupture as suggested by POCUS in the ED. Surgical repair was performed, and the patient was discharged home five days later to continue care on an outpatient basis. At the postoperative review on day 14, there were no reported complications, and the patient was in remarkable health.

## Discussion

DH may be congenital, acquired, or caused by penetrating or non-penetrating trauma. Most acquired DHs result from non-penetrating injuries to the chest or upper abdominal wall [5,7]. Although DH is typically not considered life-threatening during the initial trauma survey, it may pose risks during resuscitation [3], making early recognition important. A diagnosis of traumatic DH may be suspected based on a thorough patient history and interpretation of the mechanism of injury. However, the diagnosis may be delayed, sometimes made months or even years after the initial trauma [1].

Several diagnostic methods have been described for identifying traumatic DH, including roentgen studies with contrast agents such as Gastrografin^®^ [7], the presence of a nasogastric tube coiled above the diaphragm within the pleural cavity [6], and the appearance of bowel loops or a gastric bubble within the pleural cavity on plain chest radiography [8]. Diagnosis may be further delayed due to the limited sensitivity of clinical and radiographic evaluations [9], a challenge that is often compounded in LMICs, where immediate access to advanced imaging technologies such as CT or portable X-ray scanners may be unavailable.

Bothwell et al. (2011) reported that the sensitivity of initial plain radiographs and CT scans for detecting DH is approximately 28-68% and 50-54%, respectively. Although imaging modalities like CT may not always be accessible, the use of POCUS can expedite evaluation and diagnosis of diaphragmatic rupture in trauma settings. In the late 20th century, Kim et al. described sonographic features of traumatic DH as including a floating or non-visualized diaphragm and subphrenic fluid collections [10]. Other studies have identified abnormal diaphragmatic excursions and the direct visualization of bowel loops above the diaphragm with active peristalsis as indicative of DH on ultrasound [3]. A 2010 case report by Gangahar and Doshi also described the non-visualization of the spleen and heart in the POCUS diagnosis of diaphragmatic rupture [6].

In the present case, POCUS findings that supported a diagnosis of traumatic DH included the non-visualization or absence of the left hemidiaphragm using both high- and low-frequency probes, absence of the spleen and kidney in the left upper quadrant, and the presence of bowel loops exhibiting peristalsis adjacent to the lung parenchyma, visualized between the fifth and seventh ribs during respiratory excursions.

## Conclusions

While advanced imaging modalities such as CT scans can aid in the rapid diagnosis of traumatic DH in high-resource settings, access to such tools in LMICs is often limited. This case report demonstrates that POCUS serves as a valuable, inexpensive, safe, and readily available bedside diagnostic tool for traumatic DH in resource-limited EDs. The key POCUS findings in this case - including the non-visualization of the left hemidiaphragm, absent spleen and left kidney, and bowel loops with visible peristalsis adjacent to lung parenchyma - were instrumental in early and accurate diagnosis. These findings prompted a confirmatory chest X-ray, which revealed characteristic features of DH and enabled timely surgical intervention. This case highlights the potential of POCUS to significantly improve outcomes for trauma patients in LMICs by facilitating the prompt identification and management of this often occult and life-threatening condition.
